# Editorial: Elevating Sport Performance to New Heights With Innovative ‘Live Low – Train High’ Altitude Training

**DOI:** 10.3389/fspor.2020.00108

**Published:** 2020-08-27

**Authors:** Olivier Girard, Paul S. R. Goods, Franck Brocherie

**Affiliations:** ^1^School of Human Sciences, Exercise and Sport Science, University of Western Australia, Perth, WA, Australia; ^2^Western Australian Institute of Sport, Perth, WA, Australia; ^3^Laboratory Sport, Expertise and Performance, EA 7370, French Institute of Sport (INSEP), Paris, France

**Keywords:** altitude training, hypoxia, repeated-sprint training in hypoxia, local hypoxia, sprint interval training, normobaric hypoxia

## Historical Perspective

Traditional altitude training, where athletes are exposed to chronic hypoxia for several weeks as a means of additional physiological stimulus, has a long-standing history (Owen, 1974). The prevailing view with such an approach is that it elevates red blood cell count, increasing the amount of hemoglobin available to ferry oxygen (O_2_) from lungs to muscles, and eventually boost physical performance (Chapman et al., 2014). Debate into the effectiveness of altitude training continues with some authors strongly believing it increases endurance performance (Millet et al., 2019), while others argue that the effects of chronic hypoxia are not conclusive (Robach et al., 2012). Modalities employing intermittent hypoxia for shorter durations (minutes to hours) are also popular (McLean et al., 2014), while only cause minor disruptions to the usual athlete daily lifestyle and training routine.

“Live Low-Train High” (LLTH) interventions have been implemented by Soviet scientists as early as in the 1930s (Sirotinin, 1940). Using altitude chambers or inhaled gas, LLTH methods were first implemented for altitude pre-acclimatization of pilots who flew in open cockpits at 5,000–6,000 m and for the treatment of a variety of clinical disorders (Serebrovskaya, 2002). For long, LLTH methods included two main hypoxic modalities corresponding to intermittent hypoxic exposure (IHE) at rest or combined with exercise (intermittent hypoxic training: IHT) (Wilber, 2007). The consensus is that the use of IHE does not increase sea-level performance (Bärtsch et al., 2008), while the effects of IHT with respect to improving exercise capacity also remain elusive (Faiss et al., 2013). Our understanding of successful LLTH altitude training methods for athletes has significantly developed over the last decade (McLean et al., 2014). New technological advancements have prompted the development of innovative LLTH interventions, as summarized in an updated panorama of LLTH altitude training methods Girard et al..

## This Research Topic

The repeated-sprint training in hypoxia (RSH) paradigm requires the completion of maximal, short duration (typically ≤30 s) efforts interspersed with incomplete recovery periods (≤60 s) in hypoxic environment (Brocherie et al., 2017). This approach is now recognized as one LLTH method particularly useful for improving repeated-sprint performance in a wide range of sports, with the great majority of RSH studies having used moderately-trained individuals (Brocherie et al., 2017). For effective translation of sport science research to the field, it is crucial that research becomes “*athlete and coach problem solving focused”* (Fullagar et al., 2019). Until now, our understanding of how RSH methods are effectively implemented in high-performance settings (i.e., during specific periods of the season) is limited (Brechbuhl et al., 2018; Beard et al., 2019). In International field hockey players (Malaysia national team), a 6-week “in season” running-based RSH programme improved the succession of maximal treadmill efforts performed in hypoxia (James and Girard). In one case study, Faiss and Rapillard also reported the benefits of 150 repeated sprints at a simulated altitude of 3,300 m over 10 days in a professional cyclist. Advancing our understanding of RSH-induced adaptations also requires relevant research in sport-specific ecological test settings to be conducted. In this context, the effects of a 4-wks in-water swimming specific RSH program on freestyle swimming performance have been evaluated by Camacho-Cardenosa et al., but failed to induce further benefits than similar training in normoxia, possibly due to the selection of a sub-optimal hypoxic dose.

Other systemic or local LLTH methods based on the repetition of “all-out” efforts can be used to induce a potent physiological stimulus, up-regulate signaling pathways and eventually maximize performance outcomes. For instance, when hypoxia is induced by voluntary hypoventilation at low lung volume (VHL) (Trincat et al., 2017), RSH can also ameliorate performance compared to training with unrestricted breathing, as demonstrated by Lapointe et al. in basketball players. These authors reported that, after 8 RSH-VHL sessions including changes of direction, gains may be attributed to greater muscle reoxygenation, enhanced muscle recruitment strategies, and improved K^+^ regulation to attenuate the development of muscle fatigue, especially in type-II muscle fibers. Sprint-interval training (SIT) involves the repetition of long (~30 s) “all-out” efforts and is an effective training strategy for upregulating mitochondrial biogenesis and exercise capacity (Gibala and Hawley, 2017); however, the effect of additional hypoxia on these responses is uncertain. Takei et al. made the interesting observation that while six sessions of repeated Wingates, performed 3 times per week over 2 weeks, in either hypoxia or normoxia led to similar performance gains, more favorable blood lactate responses occurred in O_2_-deprived conditions. Finally, when compared to SIT alone, eight sessions of SIT immediately preceded by three cycles of bilateral occlusions (ischemic preconditioning: IPC) induce greater adaptations in cycling endurance performance that may be related to muscle perfusion and metabolic changes (Paradis-Deschênes et al.).

Across the Globe, an ever growing number of academic institutions, specialized clinics/hospitals (e.g., Aspetar Hospital), national sport institutes (e.g., Western Australia Institute of Sport), professional clubs (e.g., Manchester City Football Club) or national federations (e.g., France Rugby), and private gyms (e.g., The Altitude Centre) are now equipped with altitude simulation facilities. However, innovative LLTH methods such as resistance training in hypoxia can also be implemented in high performance centers located at moderate natural altitude ranging 1,800–2,400 m (e.g., Font Romeu in France or Sierra Nevada in Spain). In testing the viability of using mean propulsive velocity to adjust the load in the countermovement jump at a terrestrial altitude of 2,320 m, Rodríguez-Zamora et al. indicated that power-oriented exercises using intermittent hypobaric hypoxia allow athletes to lift higher loads, also evoking higher ratings of perceived exertion than at sea level.

## Moving Forward

Although an extensive number of LLTH studies have been published so far, several issues regarding the implementation of many innovative methods described in the updated panorama that we proposed (Girard et al.) and their physiological and performance consequences remain unresolved. The effectiveness of pre-acclimatization strategies for high altitude exposure should be explored (Fulco et al., 2013). Moreover, very few studies have considered the de-acclimatization period or how LLTH methods can be used to extend the benefits associated with chronic hypoxia (Hamlin et al., 2017) and determined how various normobaric and hypobaric hypoxic methods can be best combined [e.g., live high train low and high; (Brocherie et al., 2015)]. Finally, some practitioners are starting to include hypoxic conditioning/rehabilitation for injured athletes or patients who cannot tolerate the mechanical stress associated with higher-intensity exercise (Girard et al., 2017). Future studies are needed to refine the exercise characteristics and hypoxic dose needed to reduce the load while achieving a desired physiological response.

## Author Contributions

All authors listed have made a substantial, direct and intellectual contribution to the work, and approved it for publication.

## Conflict of Interest

The authors declare that the research was conducted in the absence of any commercial or financial relationships that could be construed as a potential conflict of interest.
